# Assessment of pharmacokinetic compatibility of short acting CDRI candidate trioxane derivative, 99–411, with long acting prescription antimalarials, lumefantrine and piperaquine

**DOI:** 10.1038/srep17264

**Published:** 2015-11-25

**Authors:** Isha Taneja, Kanumuri Siva Rama Raju, Sheelendra Pratap Singh, Muhammad Wahajuddin

**Affiliations:** 1 Academy of Scientific and Innovative Research, New Delhi, India; 2 Pharmacokinetics and Metabolism Division, CSIR- Central Drug Research Institute, Lucknow-226031, India; 3 Analytical Chemistry Division, CSIR-Indian Institute of Toxicology Research, Lucknow-226001, India

## Abstract

The pharmacokinetic compatibility of short-acting CDRI candidate antimalarial trioxane derivative, 99–411, was tested with long-acting prescription antimalarials, lumefantrine and piperaquine. LC-ESI-MS/MS methods were validated for simultaneous bioanalysis of lumefantrine and 99–411 and of piperaquine and 99–411 combinations. The interaction studies were performed in rats using these validated methods. The total systemic exposure of 99–411 increased when administered with either lumefantrine or piperaquine. However, co-administration of 99**–**411 significantly decreased the systemic exposure of piperaquine by half-fold while it had no effect on the kinetics of lumefantrine. 99–411, thus, seemed to be a good alternative to artemisinin derivatives for combination treatment with lumefantrine. To explore the reason for increased plasma levels of 99–411, an *in situ* permeability study was performed by co-perfusing lumefantrine and 99–411. In presence of lumefantrine, the absorption of 99–411 was significantly increased by 1.37 times than when given alone. Lumefantrine did not affect the metabolism of 99–411 when tested *in vitro* in human liver microsomes. Additionally, ATPase assay suggest that 99–411 was a substrate of human P-gp, thus, indicating the probability of interaction at the absorption level in humans as well.

Malaria is an infective disease with ongoing transmission in 97 countries and territories. In 2013, there were 198 million reported cases of malaria leading to an estimated 584,000 deaths worldwide. Malaria caused 453,000 deaths among children under the age of five, 437,000 of them occurring in Africa. In 2014, approximately 1.2 billion people were estimated to be at high risk of malaria. WHO currently recommends the use of combination drug therapy (especially, artemisinin based combination therapy (ACT)) for treating all cases of uncomplicated malaria infection. The available ACT treatment options are: artemether/lumefantrine (AL); dihydoartemisinin/piperaquine (DP); artesunate/amodiaquine (ASAQ); artesunate plus sulfadoxine/pyrimethamine (in areas where SP is still efficacious); artesunate/mefloquine (in areas with moderate transmission). In accordance with WHO guidelines, 79 out of 87 *P. falciparum* endemic nations have adopted ACTs as the first-line treatment^1^. AL is the most widely employed ACT worldwide while DP is the latest addition to the ACT armor. Recent studies have shown DP to be both clinically and economically superior to AL. In longitudinal follow-ups, DP has also been found to have a longer post-treatment prophylactic effect than AL (29.4 days vs 13.8 days), thus, reducing the probability of recrudescence^2^,^3^,^4^.

Recently, artemisinin resistant *P. falciparum* has been detected in five countries in the Greater Mekong subregion, namely, Cambodia, the Lao People’s Democratic Republic, Myanmar, Thailand and Viet Nam. Mutations at the residues L263 and S769 close to the binding site of the enzyme PfATP6 (a SERCA-like ATPase found in parasite’s cytoplasm) have been implicated in artemisinin resistance^5^. More recently, specific mutations in Kelch 13 (K13)-propeller domain, associated with delayed parasite clearance, was identified as a molecular marker of artemisinin resistance. In areas along Cambodia-Thailand borders, multidrug resistant *P. falciparum* has also been detected that is resistant to most presently available antimalarial drugs^1^. Current efforts have been directed to tackle the major concern of multidrug resistance. Polymorphism in *P. falciparum* chloroquine ressistance transporter (pfcrt) and *P. falciparum* multidrug resistance 1 (pfmdr1) genes have been associated with reduced sensitivity to most anitmalarials such as lumefantrine (LUME) and amodiaquine. The presence of pfmdr1 N86, D 1246 and pfcrt K76 alleles and an increased pfmdr1 copy number have been implicated as independent risk factors for recrudescence following AL treatment while pfmdr1 86Y, 1246Y and pfcrt 76T alleles have been associated with decreased sensitivity towards ASAQ and DP treatments. This alternative selection of parasitic alleles by the partner drugs could be beneficial in choosing the appropriate ACT in regions with differing pfcrt and pfmdr1 polymorphisms^6^,^7^,^8^,^9^.

The increasing prevalence of multidrug resistance *P. falciparum* and the high cost of artemisinin derivatives have propelled investigation in search of newer alternatives. In the quest to develop compounds that have improved oral bioavailability, low toxicity and are more affordable for the treatment of complicated and severe malaria, CSIR-Central Drug Research Institute (CDRI) (India) has developed promising trioxane antimalarial compounds in its drug discovery program^10^,^11^. These antimalarials are considered as a new cost effective alternative to the existing class of artemisinin drugs. CDRI 99–411 is among the most promising antimalarial trioxanes currently under clinical development. It is a highly efficacious, orally active, fast-acting antimalarial that has been found safe during regulatory pharmacology and toxicity studies. Since, it has been proposed as a substitute for shorter treatment regimen, it requires a suitable long-acting partner drug for its clinical utility. Thus, the aim of the present study is to explore the pharmacokinetic interaction potential of 99–411 with the first-line antimalarial drugs, LUME and piperaquine (PPQ) and to investigate the prospective of 99–411 as a short acting partner drug for these antimalarial drugs for useful combination therapy. Chemical structures of these compounds are shown in Fig. 1.

## Results

### LC–MS/MS optimization and method validation

Several solvents combinations, solvent compositions and column types were investigated. Waters XBridge C18 column (4.6 × 50 mm, 5.0 μm) with mobile phase consisting of acetonitrile: methanol (50:50, v/v) and 10 mM ammonium formate buffer (pH 5) in the ratio of 90:10 (v/v) at a flow rate of 0.650 mL/min provided the best compromise between selectivity and speed of analysis for LUME and 99–411 combination. The retention time of LUME and 99–411 was 3.81 and 2.09 min, respectively. For PPQ and 99–411 combination, Supelco Discover C18 column (4.6 × 50 mm, 5.0 μm) with mobile phase consisting of acetonitrile: methanol (50:50, v/v) and 10 mM ammonium formate buffer (pH 4.5) in the ratio of 90:10 (v/v) at a flow rate of 0.9 mL/min was used. The retention time of PPQ was 4.29 min and 99–411 was 3.91 min. In order to optimize ESI conditions for LUME, PPQ, 99–411 and halofantrine (internal standard, IS), quadrupole full scans were carried out in positive ion detection mode. During a direct infusion experiment, the mass spectra for LUME, PPQ, 99–411 and IS revealed peaks at m/z 529, 535.2, 412.5 and 502 respectively as protonated molecular ions [M + H]^+^. Following MRM scans, m/z 529 precursor ion [M + H]^+^ to the m/z 511.3 product ion for LUME, m/z 535.2 precursor ion [M + H]^+^ to the m/z 287 product ion for PPQ, m/z 412.5 precursor ion [M + H]^+^ to the m/z 185.4 product ion for 99–411 and m/z 502 precursor ion [M + H]^+^ to the m/z 142.2 product ion for IS were used for the quantitation purpose.

**Figure 2:**
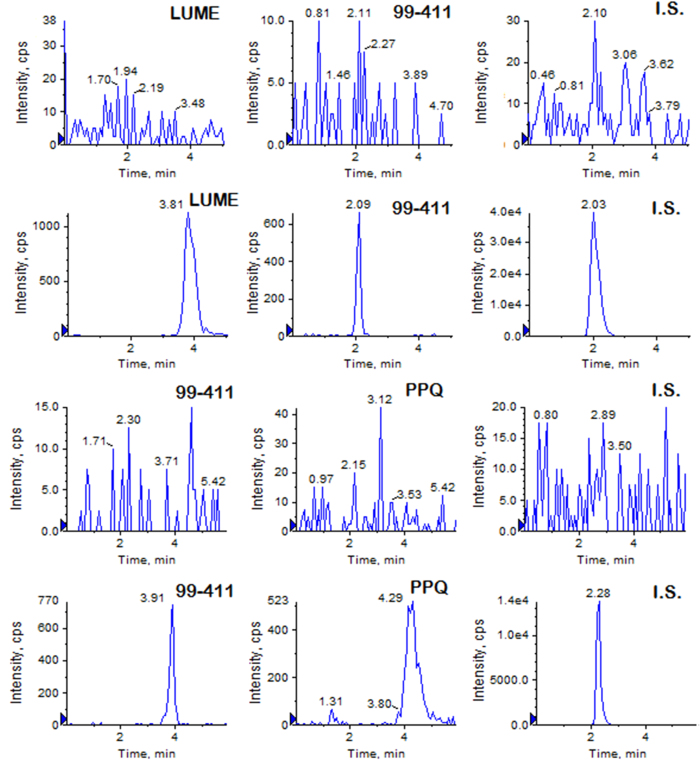
Typical MRM chromatograms of LUME and 99–411 in rat plasma (A) a drug free plasma, (B) drug free plasma spiked with LUME and 99–411 at LLOQ (3.9 ng/mL and 7.8 ng/mL respectively) and IS; and 99–411 and PPQ in rat plasma (C) a drug free plasma, (D) drug free plasma spiked with PPQ and 99–411 at LLOQ (3.9 ng/mL and 7.8 ng/mL respectively) and IS.

Protein precipitation was chosen as the extraction technique. Acetonitrile was chosen as the extraction solvent because of higher extraction efficiency for LUME, 99–411 and IS, and much cleaner samples than other solvents. The extraction recoveries of the LUME and 99–411 ranged from 98.65 to 103.54%, and the extraction recovery of the internal standard was 91.89%. The extraction recoveries of the PPQ and 99–411 ranged from 94.63 to 109.56%, and the extraction recovery of the internal standard was 93.26%. The ion suppression or enhancement by plasma was less than 8% for the analytes and IS which demonstrated that the matrix effects do not cause quantitation bias. Method specificity was confirmed by the lack of any interference from plasma at the LC peak region of the analytes as shown in Fig. 2.

The plasma calibration curve was linear within the concentration range of 3.9–500 ng/mL for LUME and PPQ and 7.8–500 ng/mL for 99–411. The average regression (n = 3) was found to be ≥0.998. The % accuracy and precision observed for the mean of back-calculated concentrations for three calibration curves was within acceptable limits for both the combinations (details provided as supplementary data). Accuracy and precision data for intra- and inter-day plasma samples are presented in Tables 1 and 2. The assay values on both the occasions (intra- and inter-day) were found to be within the accepted variable limits.

The % accuracy of diluted QCs was in the range of 94.28 to 104.66; while % precision values ranged from 2.41 to 8.62 for both the analytes, thus, confirming the validity of dilution integrity. For the series of stability tests performed, the accuracy of observed concentrations for LUME, PPQ and 99–411 at 10 and 400 ng/mL samples deviated within 91.26–106.75% while the precision ranged within 2.15–10.84% (data provided in supplementary information).

### Interaction Studies

#### Interaction study of LUME with 99–411 and vice versa

**Figure 3:**
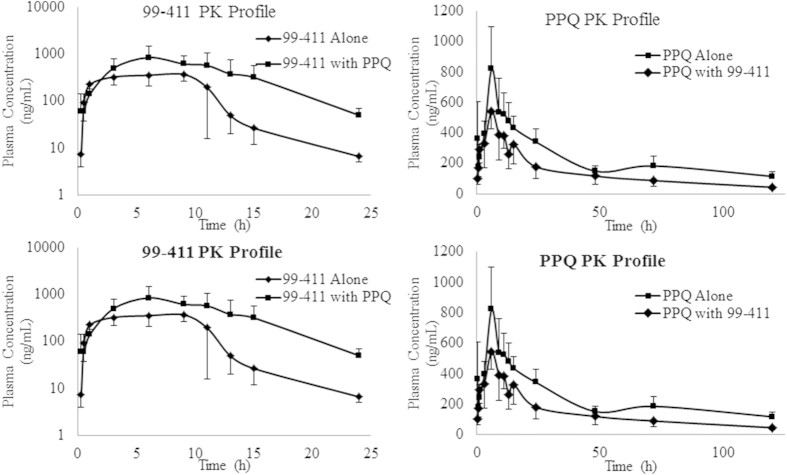
Plots of mean plasma concentration vs time of (A) LUME and 99–411 and; (B) PPQ and 99–411 following oral administration either alone or in combination (mean ± S.D.; n = 5).

The mean plasma concentration– time profiles of LUME (10 mg/kg) and 99–411 (70 mg/kg) administered orally either alone or in combination in rats are shown in Fig. 3. Table 3 summarizes the pharmacokinetic parameters of LUME and 99–411. 99–411 co-administration had no significant effect on pharmacokinetics of LUME. The presence of LUME, however, significantly increased the area under the curve, AUC_0-∞_, (493.06%; p = 0.0001) and observed maximum concentration, C_max_, (490.90%; p = 0.0042) of orally administered 99–411. Consequently, the relative bioavailability (RB%) of 99–411 in the presence of LUME is remarkably increased (493.06%; p = 0.0001) compared to the 99–411 alone. The time required to reach maximum systemic concentration (T_max_) was not significantly altered by LUME. 99–411 clearance (Cl/F) was decreased by 82.52% and the apparent volume of distribution (Vd/F) decreased by 93.32% with LUME co-administration; this resulted in a significant decrease in plasma t_1/2_ (2.13 h versus 4.85 h; p = 0.0207) since decrease in Vd/F was 2 fold higher than decrease in CL/F.

#### Interaction study of PPQ with 99–411 and vice versa

The mean plasma concentration– time profiles of PPQ (70 mg/kg) and 99–411 (70 mg/kg) administered orally either alone or in combination in rats are shown in Fig. 3 and their pharmacokinetic parameters in Table 4. The presence of 99–411 significantly (p < 0.05) decreased the AUC_0-∞_ (61.39%; p = 0.0018) and C_max_ (61.67%; p = 0.0154) of orally administered PPQ. Consequently, the relative bioavailability (RB%) of PPQ in the presence of 99–411 is remarkably decreased (61.39%; p = 0.0018) compared to the PPQ alone. The T_max_ was not significantly altered by 99–411. PPQ clearance (Cl/F) was increased by 41.94% and Vd/F increased by 194.49% with 99–411 co-administration; this resulted in a significant increase in plasma t_1/2_ (41.67 h versus 21.44 h; p = 0.0070) since increase in Vd/F was 4.6 fold higher than increase in CL/F. The presence of PPQ significantly increased the AUC_0-∞_ (196.11%; p = 0.0003) and C_max_ (202.28%; p = 0.0066) of orally administered 99–411. Consequently, the relative bioavailability (RB%) of 99–411 in the presence of PPQ is remarkably increased (196.11%; p = 0.0003) compared to the 99–411 alone. The T_max_ was not significantly reduced by PPQ. 99–411 clearance (Cl/F) was decreased by 56.06% and Vd/F decreased by 72.95% with 99–411 co-administration. As both CL/F and Vd/F were decreased, there was no significant effect on t_1/2_ by co-administration of PPQ (3.55 h versus 4.85 h).

#### *In situ* permeability study of LUME and 99–411

An *in situ* intestinal perfusion study of LUME and 99–411 was performed by co-perfusing both the analytes. The effective permeability of 99–411 significantly increased from 7.05 ± 0.58 × 10^−5^ cm/sec to 9.68 ± 0.23 × 10^−5^ cm/sec (p < 0.05) in presence of LUME while that of lumefantrine was not significantly altered (4.03 ± 0.49 × 10^−5^ cm/sec control vs 4.68 ± 1.37 × 10^−5^ cm/sec co-perfused) in the presence of 99–411. Thus, this gives useful insights as to what could be a possible mechanism for the increased total plasma concentration of 99–411.

#### Human P-gp ATPase assay

**Figure 4:**
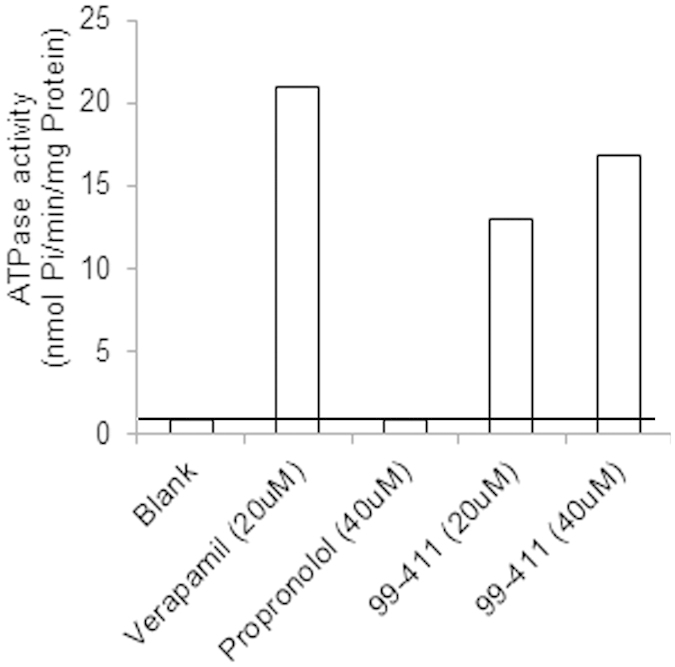
Human p-gp ATPase activities of verapamil, propranolol and 99–411. Data are shown as the mean of three independent experiments.

The ATPase activity assay was performed to assess the affinity of 99–411 for human P-gp in comparison to verapamil (positive control), and propranolol (negative control). Figure 4 shows the vanadate-sensitive ATPase activities of these compounds. 99–411 at 20 and 40 μM showed 14.63- and 18.87-fold increases in basal activity while Verapamil at 20 μM showed 23.47 fold increase when compared to that for the drug-free membrane. The basal activity in the presence of the negative control (propranolol) was only 0.96.

#### *In vitro* metabolic stability study

The *in vitro* intrinsic clearance (*Cl*_*int*_) measures the metabolic activity of microsomal proteins towards a substrate. It was found to be 1.38 ± 0.04 μL/min/mg of 99–411 without lumefantrine while in presence of lumefantrine, it was found to be 1.83 ±  0.31 μL/min/mg. The difference between the two values was statistically insignificant (P value = 0.06) suggesting that lumefantrine does not interfere with the metabolism of 99–411.

## Discussion

The antimalarial combination therapy is based upon the synergistic/additive of two or more drugs to improve therapeutic efficacy and/or delay the development of resistance. As per the WHO guidelines, for antimalarial combination therapy, the partner drugs should have independent modes of action on different biochemical targets in the parasite, should be safe when administered as combination and should be cost effective. LUME and PPQ are the current long acting partner drugs for the most widely used first line ACTs: AL and DP. These two treatment regimens have been observed to select alternate resistant mutations, thus, permitting their preferable use in regions with different epidemiological situations. Thus, LUME and PPQ were investigated for combination therapy with the CDRI candidate trioxane derivative, 99–411. In the present study, 99–411 was found to have a mean half-life of 2.99 h which shows it to be a short-acting compound. Its systemic C_max_ was reached within 30 min which suggests that it should be rapidly-acting therapeutic agent since the target of action of antimalarials is in blood. LUME and PPQ, on the other hand, showed mean half-lives of 46.94 and 21.44 h, respectively, which proves them to be long-acting as has also been reported earlier^12^,^13^. Thus, the combination of 99–411 with either LUME/PPQ fulfills WHO criteria of a short and a long-acting partner. To get an understanding on the compatibility of these combinations, their pharmacokinetic interaction studies were performed. From the two interaction studies, it was observed that while the systemic exposure of 99–411 increased with the co-administration of both LUME and PPQ, 99–411 co-administration resulted in decreased systemic levels of PPQ. Thus, the pharmacodynamics outcome of PPQ/99–411 combination could be compromised and may even result in therapy failure due to the reduced PPQ systemic concentrations. Also, since PPQ is the long acting partner, its increased clearance could lead to more probability of recrudescence and reinfection. Hence, the better combination out of the two tested treatments is LUME/99–411 for which the systemic exposure of LUME remained unaltered upon 99–411 co-administration. The increased systemic levels of 99–411 in combination with either LUME or PPQ suggest a possible dose reduction for achieving the same efficacy as when administered alone. Also, for combination with PPQ, the dosage of PPQ might be required to be re-evaluated for achieving the desired therapeutic outcome. We further explored the reasons for the increased 99–411 biological levels by investigating the effect of LUME on the absorption of 99–411. The permeability of 99–411 significantly increased by 1.37 times in presence of LUME. LUME has been reported to be P-gp substrate^14^. Since, 99–411 is also a BCS class II compound, there are chances that it may also be a P-gp substrate which may explain its observed increased intestinal permeability on co-administration with LUME. To address this, we performed an ATPase assay using human P-gp membrane at two different concentrations of 99–411 (20 or 40 mM) which suggested that 99–411 is a substrate of human P–gp. Since, LUME has been reported to be a substrate of human P-gp also; the findings of this study could be assumed to reproduce in humans as well^14^. Additionally, both LUME and 99–411 have been reported to be CYP3A4 substrates^15^,^16^,^17^. This may cause competitive inhibition of metabolism of both the drugs. Since, 99–411 is a shorter acting and fast metabolizing compound than LUME, the effect of CYP inhibition would be more evident on 99–411 metabolism than on LUME metabolism which is eliminated more slowly. This may also be a reason for increased systemic levels of 99–411 upon LUME co-administration. Thus, we tested the effect of lumefantrine on the CYP-mediated metabolism of 99–411 *in vitro* in human liver microsomes. However, no significant difference was found in the *Cl*_*int*_ values of 99–411 in presence and absence of lumefantrine. The results of the *in vitro* interaction study in human liver microsomes suggest insignificant interaction potential of LUME and 99–411 due to the alteration of drug metabolizing enzyme activity. However, absorption related interaction involving P-gp could occur at the intestinal level as seen for rats in this study. To extrapolate the results to predict *in vivo* interaction potential of 99–411 and LUME in humans, the human equivalent dose of 99–411 was calculated to be 11.29 mg/kg based on 70 mg/kg rat dose used in the present study. For lumefantrine, the established human dose is 480 mg per dose^18^. The human intestinal concentrations that would be reached (calculated as molar dose/250 mL) by both the compounds would be higher than the concentrations tested positive *in situ* using SPIP rat model. This, in combination with the fact that both LUME and 99–411 have affinity for human P-gp, suggest that there is a high predictability of finding similar interaction, as seen in rats, in humans as well.

The present study provides a preliminary groundwork for dosage adjustment for further pharmacodynamic studies of the proposed combinations. The results show that there are potential chances of pharmacokinetic interaction for both the combinations: short acting 99–411 and long acting LUME/PPQ. LUME co-administration significantly enhanced the exposure of 99–411, which may be beneficial for the therapeutic efficacy. However, PPQ with 99–411 was found to have reduced oral exposure which may compromise the pharmacodynamic outcome upon per-oral administration. Thus, out of the two interactions evaluated pharmacokinetically, LUME with 99–411 seems to have therapeutic merit in the present study. These results of the present study should be taken in to consideration while designing clinical proof of concept pharmacodynamic studies and while designing dosage regimen. Clinically, both LUME and 99–411, being substrates of P-gp and CYP3A4, could have drug interaction potential with other P-gp and/or CYP3A4 substrates and inhibitors. The fact should, especially, be considered while coadministering this combination with HIV protease inhibitors as many of them have been reported to be CYP3A4 and P-gp inhibitors and/or substrates. Malaria and HIV are co-endemic diseases, thus, there is a high probability of co-prescription of antimalarial and anti-HIV drugs. Also, pharmacogenetic differences leading to differences in metabolism of these CYP3A4/P-gp substrates and inhibitors could cause alterations in LUME and 99–411 systemic levels and thus, treatment outcome. For LUME, it was observed that the patients receiving concomitant efavirenz therapy had significantly lower day 7 LUME plasma levels associated with CYP2B6*6 genotype; while concomitant nevirapine treatment had no effect on day 7 LUME plasma concentrations. It was also observed that in patients having defective P-gp genotype (*ABCB1 3435* *TT*) variant, nevirapine co-treatment was associated with higher LUME plasma levels^19^. Further, genetic variations in CYP3A4 could alter LUME and 99–411 systemic concentrations by directly altering their metabolism. A high allelic frequency of mutated CYP3A4*1B and low frequency of the wild type CYP3A5*1 was associated with altered metabolism of LUME and artemether, causing significantly lower cure rates by AL treatment in Cambodian patients^20^. Thus, additional pharmacodynamic, mechanistic and regulatory studies need to be performed and pharmacokinetic data from *in vivo* malaria model and higher experimental animals need to be evaluated for a better and more informed decision. The efficacy data of the combination is required to be correlated to the pharmacokinetic data to understand its PK/PD relationship.

## Material and Methods

### Chemicals and reagents

LUME, PPQ and halofantrine (internal standard, IS) were a generous gift from Ipca Laboratories Ltd. (Mumbai, India). 99–411 was synthesized at the Medicinal Chemistry Division of CSIR-Central Drug Research Institute (CDRI) (Lucknow, India). Human P-gp ATPase assay kit, human P-gp membrane and human liver microsomes were purchased from BD Gentest^®^ (Woburn, MA). Ammonium formate AR was purchased from E Merck Limited (Mumbai, India). HPLC grade acetonitrile, methanol, polyethylene glycol (PEG400) and sodium carboxy methyl cellulose (CMC) were purchased from Sigma Aldrich Ltd (St Louis, USA). Urethane was purchased from Thermo Fisher Scientific India Pvt. Ltd. (Mumbai, India). Ultrapure water was obtained from a Sartorious Arium 611 system. Heparin sodium injection I.P. (1000 IU/mL, Biologicals E. Limited, Hyderabad, India) was purchased from local pharmacy.

Blank, drug free plasma samples were collected from adult, healthy male *Sprague–Dawley* (SD) rats at the Division of Laboratory Animals (DOLA) of Central Drug Research Institute (Lucknow, India). Plasma was obtained by centrifuging the heparinised blood (25 IU/mL) at 2000 × g for 10 min at 20 °C. Prior approval from the Institutional Animal Ethics Committee (IAEC) was sought for maintenance, experimental studies, euthanasia and disposal of carcass of animals.

### Instrumentation and chromatographic conditions

HPLC system consists of Series 200 pumps and auto sampler with temperature controlled Peltier-tray (Perkin- Elmer instruments, Norwalk, USA) was used to inject 10 μL aliquots of the processed samples. For LUME and 99–411 combination, on a Waters XBridge C18 column (4.6 × 50 mm, 5.0 μm) was used and the system was run in isocratic mode with mobile phase consisting of acetonitrile: methanol (50:50, v/v) and 10 mM ammonium formate buffer (pH = 5.0) in the ratio of 90:10 (v/v) at a flow rate of 0.650 mL/min. For PPQ and 99–411 combination, samples were analysed on a Supelco Discovery C18 column (4.6 × 50 mm, 5.0 μm). The system was run in isocratic mode with mobile phase consisting of acetonitrile: methanol (50:50, v/v) and 10 mM ammonium formate buffer (pH = 4.5) in the ratio of 90:10 (v/v) at a flow rate of 0.90 mL/min. Mobile phase was duly filtered through 0.22 μm Millipore filter (Billerica, USA) and degassed ultrasonically for 15 min prior to use.

Mass spectrometric detection was performed on an API 4000 mass spectrometer (Applied Biosystems, MDS Sciex Toronto, Canada) equipped with an API electrospray ionization (ESI) source. The ion spray voltage was set at 5500 V. The instrument parameters viz., nebulizer gas, curtain gas, auxillary gas and collision gas were set at 40, 13, 50 and 10, respectively. Compounds parameters viz., declustering potential (DP), collision energy, entrance potential and collision exit potential (CXP) were 80, 33, 10, 10 V, 30, 20, 4, 10 V and 90, 33, 6, 8 V for LUME, 99–411 and IS, respectively and were 110, 42, 10, 10 V, 30, 20, 4, 10 V and 90, 33, 6, 8 V for PPQ, 99–411 and IS, respectively. Zero air was used as source gas while nitrogen was used as both curtain and collision gas. The mass spectrometer was operated at ESI positive ion mode and detection of the ions was performed in the multiple reaction monitoring (MRM) mode, monitoring the transition of m/z 529 precursor ion [M + H]^+^ to the m/z 511.3 product ion for LUME, m/z 412.5 precursor ion [M + H]^+^ to the m/z 185.4 product ion for 99–411, m/z 535.2 precursor ion [M + H]^+^ to the m/z 287 product ion for PPQ and m/z 502 precursor ion [M + H]^+^ to the m/z 142.2 product ion for IS. Quadrupole 1 and quadrupole 3 were maintained at unit resolution and dwell time was set at 400 ms. Data acquisition and quantitation were performed using analyst software version 1.4.1 (Applied Biosystems, MDS Sciex Toronto, Canada).

### Preparation of standard and quality control samples

Primary stock solutions of LUME, PPQ, 99–411 and IS were prepared by dissolving the compounds in acidified methanol (1% acetic acid) to achieve desired concentration of 1 mg/mL. Working standard solutions of all analytes and IS (50 ng/mL) were prepared by diluting an aliquot of primary stock solution with acetonitrile. Calibration standards of all analytes (3.9, 7.8, 15.6, 31.25, 62.5, 125, 250 and 500 ng/mL) were prepared by spiking 90 μL of pooled drug free rat plasma with the appropriate working standard solution of the analytes (10 μL). All the stock solutions were stored at 4 °C until analysis. Quality control (QC) samples were prepared by individually spiking control rat plasma at four concentration levels [3.9 ng/mL for LUME and PPQ and 7.8 ng/mL for 99–411 (lower limit of quantitation, LLOQ), 10 ng/mL (QC low), 100 ng/mL (QC medium) and 400 ng/mL (QC high)] and stored at −70 ± 10 °C until analysis.

### Sample preparation

A simple protein precipitation extraction method was followed. To 100 μL plasma in a tube, 200 μL of IS solution (50 ng/mL in acetonitrile), was added and vortexed for 10 min followed by centrifugation for 10 min at 15000 × g. The supernatant was separated and 10 μL was injected onto analytical column.

### Validation procedures

Validation was carried out as per US FDA bioanalytical method validation guidelines^21^. The extraction recovery of analytes, through protein precipitation extraction procedure, was determined by comparing the peak areas of extracted plasma (pre-spiked) standard QC samples (n = 6) to those of the post-spiked standards at equivalent concentrations (QC low, QC medium and QC high concentrations for the analytes and 50 ng/mL for IS). Specificity and selectivity was studied by using independent plasma samples from six different rats to investigate the potential interferences at the LC peak region for analyte and IS, using the proposed extraction procedure and chromatographic–MS conditions. Matrix effect was determined by comparing the responses of the post-extracted plasma standard QC samples (n = 6) with the response of analytes from neat standard samples, for all analytes at LLOQ^22^. Calibration curve was constructed using seven calibration standards of LUME and 99–411 and of PPQ and 99–411 (3.9, 7.8, 15.6, 31.25, 62.5, 125, 250 and 500 ng/mL) by spiking 90 μL of pooled drug free rat plasma with the appropriate working standard solution of the analytes (10 μL). The intra-day assay precision and accuracy were estimated by analyzing six replicates at four the QC levels, i.e., 3.9/7.8, 10, 100 and 400 ng/mL,for both the combinations. The inter-assay precision was determined by analyzing the four levels QC samples on three different runs. The criteria for acceptability of the data included accuracy within ± 15% deviation from the nominal values and a precision of within ± 15% relative standard deviation (R.S.D.), except for LLOQ, where it should not exceed ± 20% of accuracy as well as precision. All stability studies were conducted at two concentration levels, i.e. QC low and QC high, using six replicates at each concentration level, to establish autosampler stability (18 h), short-term stability (6 h) at room temperature (bench top, BT), long-term (LT) freezer stability at −70 ± 10 °C for at least 15 days and freeze/thaw (FT) stability up to three cycles. The dilution integrity experiment was performed to validate the dilution test to be carried out on higher analyte concentrations (above the upper limit of quantification), which may be encountered during real subject samples analysis. Dilution integrity experiments were carried out by 20 times dilution of plasma samples containing 8000 ng/mL of LUME and 99–411 and PPQ and 99–411 with blank plasma to obtain samples containing 400 ng/mL of the respective combinations.

### Interaction study of LUME and PPQ with 99–411 and vice versa

Rats were divided into five groups (n = 5, each); three control groups (LUME 10 mg/kg, oral, suspension in 0.25% Sodium CMC; PPQ 50 mg/kg, oral, solution in triple distilled water; and 99–411 70 mg/kg, oral, suspension in 0.25% Sodium CMC) and two co-administration group (70 mg/kg of oral 99–411 & 10 mg/kg of oral LUME; and 70 mg/kg of oral 99–411 & 50 mg/kg of oral PPQ). The rats were fasted overnight (14–16 h) prior to the experiment but given free access to water. Blood samples were collected from the retro-orbital plexus into heparinized microfuge tubes at 0.25, 0.50, 1, 3, 6, 9, 11, 13, 15, 24, 48, 72 and 120 h post-dosing and plasma was harvested by centrifuging the blood at 15000 × g for 10 min and stored frozen at –70 ± 10 °C until bioanalysis. Plasma data were subjected to non-compartmental pharmacokinetics analysis using WinNonlin (version 5.1, Pharsight Corporation, Mountain View, USA). The pharmacokinetic parameters were compared using student’s t test. A P-value of < 0.05 was considered significant.

The relative bioavailability (RB%) was calculated according to equation 1 as follows:


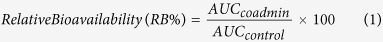


### *In situ* permeability study

Single-pass intestinal perfusion studies in rats (n = 5 per group) were performed using established methods adapted from the literature^14^,^23^. Briefly, male SD rats were fasted overnight for 12 to 16 h with free access to water and anaesthetized using an intra-peritoneal injection of urethane (1 g/kg) and placed on a heated pad to keep normal body temperature. Upon verification of the loss of pain reflex, a midline longitudinal abdominal incision was made, and the lumen of the jejunum (10 cm) was catherised and flushed with 10 ml of saline pre-warmed to 37 °C. The solution of analytes (10 μM) was prepared in perfusion buffer and passed at a flow rate of 0.2 mL/min through the lumen. After allowing 30 min to reach steady-state outlet concentrations, outlet perfusate samples were collected every 15 min for 120 min perfusion period. Phenol red was used as a marker of osmosis/zero permeability. At the end, the length of segment was measured without stretching and finally the animal was euthanized. Samples were analysed by LC-MS/MS after diluting 20 times with mobile phase.

### Human P-gp ATPase assay

A human P-gp ATPase assay was performed according to the manufacturer’s protocol. Briefly, 20 μg of human P-gp membrane (20 μL of 1 mg/mL) was preincubated at 37 °C for 5 min in a 40-μL reaction mixture with 99–411 (20 or 40 mM) in absence or presence of 300 μM sodium orthovanadate in a 96-well plate. The reaction was initiated by the addition of 20 μL of 12 mM Mg ATP solution and was terminated 20 min later by the addition of 30 μL of stop solution (10% sodium dodecyl sulfate solution). Two hundred microliters of detection reagent (8% ascorbic acid, 0.8% ammonium molybdate, 3 mM zinc acetate) was added, and the mixture was incubated at 37 °C for 20 min. The inorganic phosphate complex was detected by its absorbance at 800 nm and was quantitated by comparing the absorbance with that of phosphate standard. The validity of the assay was assessed by using verapamil and propranolol as positive and negative controls, respectively. The vanadate-sensitive ATP hydrolysis was determined by subtracting the value obtained with the vanadate-coincubated membrane fraction from that obtained with the vanadate-free membrane fraction.

### *In vitro* metabolic stability study

The *in vitro m*etabolic stability of 99–411 was performed in triplicate using glass tubes with and without lumefantrine. To each tube, 447.5 μl of phosphate buffer (0.1 M, pH 7.4) and 25 μl of microsomal protein (20 mg/ml) was added and incubated for at 37 ± 0.2 °C for 5 min. Then 2.5 μl of test compound (0.4 mM) was added. In the lumefantrine interaction setup, 2.5 μl of phosphate buffer was replaced by lumefantrine (0.4 mM). For positive control, testosterone was used as the test compound. Reaction was initiated by addition of 25 μl of NADPH (24 mM) and incubated for 0, 5, 15, 30, 45 and 60 min. In negative control, NADPH was replaced by 25 μl of phosphate buffer. Reaction was stopped by addition of 500 μl ice-cold methanol at predefined time intervals, followed by centrifugation for 15 min at 10,000 rpm. Supernatant was directly analysed by LC-MS/MS. The data was analysed using GraphPad Prism 5.0 software. Non-linear regression of percent parent remaining against time was performed. The *in vitro* intrinsic clearance (*Cl*_*int*_) was estimated using equation 2 as follows:


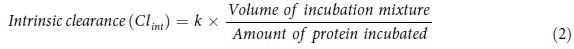


## Conclusion

Accurate and sensitive bioanalytical methods were developed and validated for simultaneous estimation of LUME and 99–411 and of PPQ and 99–411 using LC-ESI-MS/MS. Of the two combinations tested *in vivo* in rats, 99–411 and lumefantrine seems more favorable. The systemic exposure of 99–411 increased when co-administered with lumefantrine which could be due to the increased absorption of 99–411 in presence of lumefantrine. Similar interaction might also be observed in humans since ATPase assay suggest that 99–411 has affinity for human P-gp as well.

## Additional Information

**How to cite this article**: Taneja, I. *et al.* Assessment of pharmacokinetic compatibility of short acting CDRI candidate trioxane derivative, 99–411, with long acting prescription antimalarials, lumefantrine and piperaquine. *Sci. Rep.* **5**, 17264; doi: 10.1038/srep17264 (2015).

## Supplementary Material

Supplementary Information

## Figures and Tables

**Figure 1:**
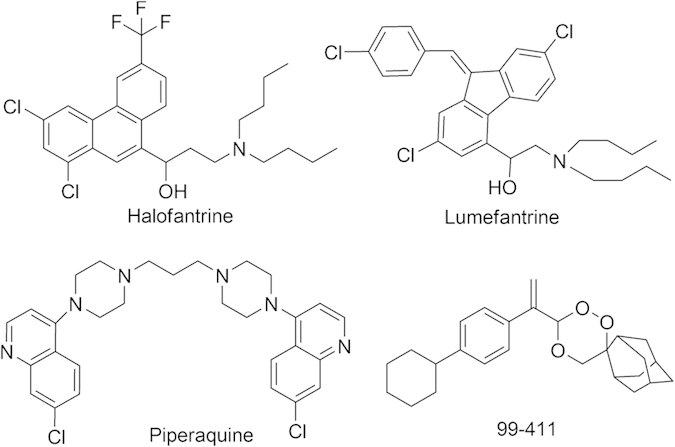
Structural representation of lumefantrine, piperaquine, halofantrine and 99–411.

**Table 1:** Intra-day and inter-day assay precision and accuracy for LUME and 99–411 in rat plasma.

	LUME (ng/mL)				99–411 (ng/mL)			
	3.9	10	100	400	7.8	10	100	400
**Intra-day (n** = **6)**								
**Day-1**								
Mean	3.97	10.14	98.82	398.33	8.11	9.83	102.33	395.50
SD	0.20	0.84	6.97	8.57	0.46	0.71	4.72	31.73
Precision^a^ (%)	5.05	8.27	7.05	2.15	5.70	7.21	4.61	8.02
Accuracy^b^ (%)	101.88	101.35	98.82	99.58	103.97	98.33	102.33	98.88
**Day-2**								
Mean	3.77	10.10	95.67	389.00	7.89	9.73	98.18	391.83
SD	0.23	0.61	4.45	12.88	0.44	0.35	6.36	22.59
Precision^a^ (%)	6.03	5.99	4.65	3.31	5.54	3.58	6.48	5.76
Accuracy^b^ (%)	96.71	101.00	95.67	97.25	100.15	97.30	98.18	97.96
**Day-3**								
Mean	3.85	10.27	101.95	406.83	7.72	9.30	94.07	430.50
SD	0.18	0.42	8.28	20.82	0.74	0.58	5.54	28.09
Precision^a^ (%)	4.68	4.04	8.12	5.12	9.52	6.26	5.89	6.53
Accuracy^b^ (%)	98.80	102.68	101.95	101.71	99.02	92.97	94.07	107.63
**Inter-day (n** = **18)**								
Mean^c^	3.87	10.17	98.81	398.06	7.91	9.62	99.80	405.94
SD	0.21	0.61	6.87	15.94	0.55	0.58	5.23	31.62
Precision^a^ (%)	5.42	5.99	6.96	4.00	6.98	6.07	5.24	7.79
Accuracy^b^ (%)	99.13	101.68	98.81	99.65	101.40	96.30	98.19	101.78

^a^Expressed as % R.S.D. = (S.D./mean) × 100.

^b^Calculated as (mean determined concentration/nominal concentration) × 100.

^c^n = 3 days with six replicates per day.

**Table 2:** Intra-day and inter-day assay precision and accuracy for PPQ and 99–411 in rat plasma.

	PPQ (ng/mL)				99–411 (ng/mL)			
	3.9	10	100	400	7.8	10	100	400
**Intra-day (n** = **6)**								
**Day-1**								
Mean	4.02	9.61	98.28	388.00	7.83	10.16	89.25	375.83
SD	0.24	0.51	2.36	20.47	0.28	0.52	3.94	18.28
Precision^a^ (%)	6.00	5.31	2.40	5.28	3.58	5.17	4.42	4.86
Accuracy^b^ (%)	103.03	96.08	98.56	96.95	100.33	101.57	89.25	93.96
**Day-2**								
Mean	3.74	9.43	95.62	375.83	8.21	10.27	94.35	412.00
SD	0.16	0.45	2.68	19.77	0.32	0.43	6.57	16.05
Precision^a^ (%)	4.16	4.79	2.80	5.26	3.94	4.14	6.96	3.89
Accuracy^b^ (%)	96.00	94.33	95.84	93.96	105.26	102.68	94.35	103.00
**Day-3**								
Mean	3.93	9.13	101.45	430.20	7.87	9.99	94.37	412.50
SD	0.16	0.45	5.61	15.79	0.29	0.40	4.63	11.50
Precision^a^ (%)	4.16	4.91	5.53	3.67	3.65	3.98	4.91	2.79
Accuracy^b^ (%)	100.64	91.32	101.45	107.55	100.94	99.88	94.37	103.13
**Inter-day (n** = **18)**								
Mean^c^	3.90	9.38	98.45	396.12	7.96	10.14	92.66	399.41
SD	0.21	0.48	4.36	29.28	0.33	0.44	5.45	23.07
Precision^a^ (%)	5.47	5.14	4.43	7.39	4.09	4.35	5.88	5.78
Accuracy^b^ (%)	99.81	93.99	98.66	98.65	102.09	101.57	92.81	98.96

^a^Expressed as % R.S.D. = (S.D./mean) × 100.

^b^Calculated as (mean determined concentration/nominal concentration) × 100.

^c^n = 3 days with six replicates per day.

**Table 3:** The pharmacokinetic parameters of LUME and 99–411 in rats (n = 5, each) All data are expressed as mean ± SD.

Parameters	Control		LUME with 99–411	
	LUME (10 mg/kg)	99–411 (70 mg/kg)	LUME (10 mg/kg)	99–411 (70 mg/kg)
**AUC**_**0-t**_ (h*μg/mL)	16.52 ± 2.96	2.99 ± 0.30	15.40 ± 3.58	17.72 ± 3.21#
**AUC**_**0-∞**_ (h*μg/mL)	17.42 ± 3.29	3.60 ± 0.28	16.51 ± 3.51	17.75 ± 3.22#
**C**_**max**_ **(μg/mL)**	1.81 ± 0.75	0.44 ± 0.06	1.74 ± 0.67	2.16 ± 0.97#
**T**_**max**_ **(h)**	3.75 ± 1.50	8.75 ± 2.06	3.60 ± 1.34	11.5 ± 3#
**V**_**d**_**/F (L/kg)**	39.44 ± 6.261	187.91 ± 78.83	46.36 ± 16.39	12.57 ± 3.70#
**CL/F (L/h/kg)**	0.59 ± 0.09	23.06 ± 2.24	0.63 ± 0.12	4.03 ± 0.64#
**t**_**1/2**_ **(h)**	46.94 ± 6.52	4.85 ± 2.08	50.75 ± 10.21	2.13 ± 0.39#
**RB (%)**	–	–	93.22	493.06#

^*^P < 0.05, vs LUME control; ^#^P < 0.05 vs 99–411 control.

**Table 4:** The pharmacokinetic parameters of PPQ and 99–411 in rats (n = 5, each) All data are expressed as mean ± SD.

Parameters	Control		PPQ with 99–411	
	PPQ	99–411	PPQ	99–411
	(50 mg/kg)	(70 mg/kg)	(50 mg/kg)	(70 mg/kg)
**AUC**_**0-t**_ (h*μg/mL)	28.09 ± 2.79	2.99 ± 0.29	16.95 ± 5.03*	6.82 ± 1.34#
**AUC**_**0-∞**_ (h*μg/mL)	31.52 ± 2.68	3.60 ± 0.28	19.35 ± 5.29*	7.06 ± 1.22#
**C**_**max**_ **(μg/mL)**	0.89 ± 0.23	0.44 ± 0.06	0.54 ± 0.11*	0.89 ± 0.27#
**T**_**max**_ **(h)**	6.60 ± 1.34	8.75 ± 2.06	6.00 ± 0.00	5.25 ± 2.87
**V**_**d**_**/F (L/kg)**	49 ± 8.38	187.91 ± 78.83	166.39 ± 71.72*	50.84 ± 8.08#
**CL/F (L/h/kg)**	1.6 ± 0.15	23.06 ± 2.23	2.73 ± 0.68*	10.133 ± 1.74#
**t**_**1/2**_ **(h)**	21.44 ± 4.39	4.85 ± 2.08	41.67 ± 11.79*	3.55 ± 0.878
**RB (%)**			61.39*	196.11#

^*^p < 0.05, vs PPQ control; ^#^p < 0.05, vs 99–411 control.
