# Supplementary material for: Assessment of pharmacokinetic compatibility of short acting CDRI candidate trioxane derivative, 99–411, with long acting prescription antimalarials, lumefantrine and piperaquine
Source: Sci Rep. 2015 Nov 25;5:17264. doi: 10.1038/srep17264 (PMC4658560; doi:10.1038/srep17264)
Supplement: Supplementary Information [file srep17264-s1.doc]

**Assessment of pharmacokinetic compatibility of short-acting CDRI candidate trioxane derivative, 99-411 with long acting prescription antimalarials, lumefantrine and piperaquine**

**Isha Taneja^1,2,+^, Kanumuri Siva Rama Raju^1,2,+^, Sheelendra Pratap Singh^3^, Wahajuddin^1,2,*^**

^1^ Academy of Scientific and Innovative Research, New Delhi, India.

^2^ Pharmacokinetics and Metabolism Division, CSIR- Central Drug Research Institute, Lucknow, India.

^3^ Analytical Chemistry Division, CSIR-Indian Institute of Toxicology Research, Lucknow, India.

*Corresponding author

Dr. Wahajuddin,

Scientist, CSIR-Central Drug Research Institute, Lucknow-226031, Uttar Pradesh, India

Tel.: +91-522-2772450/2550-4849(Office)/4850 (Lab)

E-mail address: [wahajuddin@cdri.res.in](mailto:wahajuddin@cdri.res.in), wahajuddin@gmail.com

^+^ These authors contributes equally to the work

**Table 1 Precision and accuracy data of back-calculated concentrations of calibration samples for LUME and 99-411 in rat plasma (n = 3)**

|  | **LUME** | | | |  | **99-411** | | | |
| --- | --- | --- | --- | --- | --- | --- | --- | --- | --- |
| Nominal conc. (ng/mL) | Mean | SD | Precision ^a^ (%) | Accuracy ^b^ (%) |  | Mean | SD | Precision ^a^ (%) | Accuracy ^b^ (%) |
| 3.90 | 4.01 | 0.16 | 4.04 | 102.91 |  | - | - | - | - |
| 7.80 | 7.72 | 0.27 | 3.44 | 99.02 |  | 7.68 | 0.44 | 5.66 | 98.50 |
| 15.63 | 16.53 | 0.96 | 5.81 | 105.81 |  | 15.63 | 1.05 | 6.72 | 100.05 |
| 31.25 | 28.77 | 2.25 | 7.82 | 92.05 |  | 31.03 | 2.39 | 7.69 | 99.31 |
| 62.50 | 64.80 | 0.72 | 1.11 | 103.68 |  | 63.20 | 1.91 | 3.02 | 101.12 |
| 125 | 123.00 | 1.00 | 0.81 | 98.40 |  | 127.67 | 5.51 | 4.31 | 102.13 |
| 250 | 264.33 | 13.43 | 5.08 | 105.73 |  | 273.33 | 9.02 | 3.30 | 109.33 |
| 500 | 497.33 | 12.22 | 2.46 | 99.47 |  | 478.67 | 19.14 | 4.00 | 95.73 |

^a^ Expressed as % R.S.D. = (S.D./mean) × 100.

^b^ Calculated as (mean determined concentration/nominal concentration) × 100.

**Table 2 Precision and accuracy data of back-calculated concentrations of calibration samples for PPQ and 99-411 in rat plasma (n = 3)**

| Nominal conc. (ng/mL) | **PPQ** | | | |  | **99-411** | | | |
| --- | --- | --- | --- | --- | --- | --- | --- | --- | --- |
|  | Mean | SD | Precision ^a^ (%) | Accuracy ^b^ (%) |  | Mean | SD | Precision ^a^ (%) | Accuracy ^b^ (%) |
| 3.90 | 4.03 | 0.21 | 5.18 | 103.33 |  | - | - | - | - |
| 7.80 | 7.52 | 0.33 | 4.41 | 96.41 |  | 8.06 | 0.41 | 5.14 | 103.29 |
| 15.63 | 15.90 | 0.69 | 4.36 | 101.76 |  | 15.87 | 0.26 | 1.66 | 101.55 |
| 31.25 | 31.90 | 0.72 | 2.26 | 102.08 |  | 31.75 | 1.49 | 4.70 | 101.60 |
| 62.50 | 60.17 | 1.55 | 2.58 | 96.27 |  | 60.70 | 2.48 | 4.08 | 97.12 |
| 125 | 124.67 | 2.08 | 1.67 | 99.73 |  | 121.00 | 4.00 | 3.31 | 96.80 |
| 250 | 263.67 | 13.05 | 4.95 | 105.47 |  | 261.00 | 19.00 | 7.28 | 104.40 |
| 500 | 512.00 | 14.42 | 2.82 | 102.40 |  | 518.67 | 20.01 | 3.86 | 103.73 |

^a^ Expressed as % R.S.D. = (S.D./mean) × 100.

^b^ Calculated as (mean determined concentration/nominal concentration) × 100.

**Table 3** Stability of LUME and 99-411 in rat plasma

|  | **LUME** | | | |  | **99-411** | | | |
| --- | --- | --- | --- | --- | --- | --- | --- | --- | --- |
|  | Mean^a^ | SD | Precision^b^ (%) | Accuracy^c^ (%) |  | Mean^a^ | SD | Precision^b^ (%) | Accuracy^c^ (%) |
| **10 (ng/mL)** |  |  |  |  |  |  |  |  |  |
| 0 h (for all) | 10.14 | 0.84 | 8.27 | 101.35 |  | 9.83 | 0.71 | 7.21 | 98.33 |
| 24 h (AS) | 9.98 | 0.40 | 4.05 | 98.45 |  | 9.98 | 0.40 | 4.05 | 101.47 |
| 6 h (BT) | 9.30 | 0.47 | 5.01 | 91.73 |  | 10.39 | 0.40 | 3.85 | 105.64 |
| FT-3 | 9.26 | 1.00 | 10.84 | 91.33 |  | 9.39 | 0.57 | 6.11 | 95.51 |
| 15 day at -70ºC | 9.91 | 0.42 | 4.22 | 97.80 |  | 10.19 | 0.32 | 3.10 | 103.59 |
|  |  |  |  |  |  |  |  |  |  |
| **400 (ng/mL)** |  |  |  |  |  |  |  |  |  |
| 0 h (for all) | 398.33 | 8.57 | 2.15 | 99.58 |  | 395.50 | 31.73 | 8.02 | 98.88 |
| 24 h (AS) | 398.83 | 11.69 | 2.93 | 100.13 |  | 386.83 | 22.07 | 5.70 | 97.81 |
| 6 h (BT) | 382.17 | 14.25 | 3.73 | 95.94 |  | 383.67 | 24.44 | 6.37 | 97.01 |
| FT-3 | 402.17 | 16.87 | 4.19 | 100.96 |  | 394.67 | 12.47 | 3.16 | 99.79 |
| 15 day at -70ºC | 403.50 | 8.89 | 2.20 | 101.30 |  | 392.67 | 29.19 | 7.43 | 99.28 |

^a^ Back calculated plasma concentrations (n=6)

^b^ Expressed as % R.S.D. = (S.D./mean) × 100.

^c^ Calculated as (mean determined concentration/nominal concentration) × 100.

**Table 4** Stability of PPQ and 99-411 in rat plasma

|  | PPQ | | | |  | 99-411 | | | |
| --- | --- | --- | --- | --- | --- | --- | --- | --- | --- |
|  | Mean^a^ | SD | Precision^b^ (%) | Accuracy^c^ (%) |  | Mean^a^ | SD | Precision^b^ (%) | Accuracy^c^ (%) |
| **10 (ng/mL)** |  |  |  |  |  |  |  |  |  |
| 0 h (for all) | 9.43 | 0.45 | 4.79 | 94.33 |  | 10.27 | 0.43 | 4.14 | 102.68 |
| 24 h (AS) | 9.98 | 0.40 | 4.05 | 105.78 |  | 10.43 | 0.44 | 4.20 | 101.54 |
| 6 h (BT) | 9.37 | 0.48 | 5.16 | 99.31 |  | 9.87 | 0.64 | 6.44 | 96.15 |
| FT-3 | 9.39 | 0.62 | 6.63 | 99.51 |  | 10.49 | 0.53 | 5.01 | 102.14 |
| 15 days at -70ºC | 10.07 | 0.46 | 4.59 | 106.75 |  | 9.71 | 0.44 | 4.56 | 94.56 |
| **400 (ng/mL)** |  |  |  |  |  |  |  |  |  |
| 0 h (for all) | 375.83 | 19.77 | 5.26 | 93.96 |  | 376.00 | 25.02 | 6.65 | 94.00 |
| 24 h (AS) | 385.17 | 16.44 | 4.27 | 102.48 |  | 406.83 | 14.16 | 3.48 | 98.75 |
| 6 h (BT) | 383.83 | 20.91 | 5.45 | 102.13 |  | 365.04 | 15.72 | 4.31 | 91.26 |
| FT-3 | 379.50 | 15.44 | 4.07 | 100.98 |  | 420.83 | 21.52 | 5.11 | 102.14 |
| 15 days at -70ºC | 389.33 | 12.23 | 3.14 | 103.59 |  | 397.50 | 11.67 | 2.94 | 96.48 |

^a^ Back calculated plasma concentrations (n=6)

^b^ Expressed as % R.S.D. = (S.D./mean) × 100.

^c^ Calculated as (mean determined concentration/nominal concentration) × 100.
